# Supplementary material for: Subsequent Upper Urinary Tract Carcinoma Related to Worse Survival in Patients Treated with BCG
Source: Cancers (Basel). 2023 Mar 28;15(7):2002. doi: 10.3390/cancers15072002 (PMC10092972; doi:10.3390/cancers15072002)
Supplement: Supplementary file 1 [file cancers-15-02002-s001.zip › cancers-2256756-supplementary.pdf]

Supplement Figure S1. Selection of patients. Of 3226 patients with non-muscle invasive bladder cancer treated by BCG bladder instillation therapy, we analyzed 2873 patients who recorded history with or without subsequent diagnosis with upper urinary tract carcinoma.

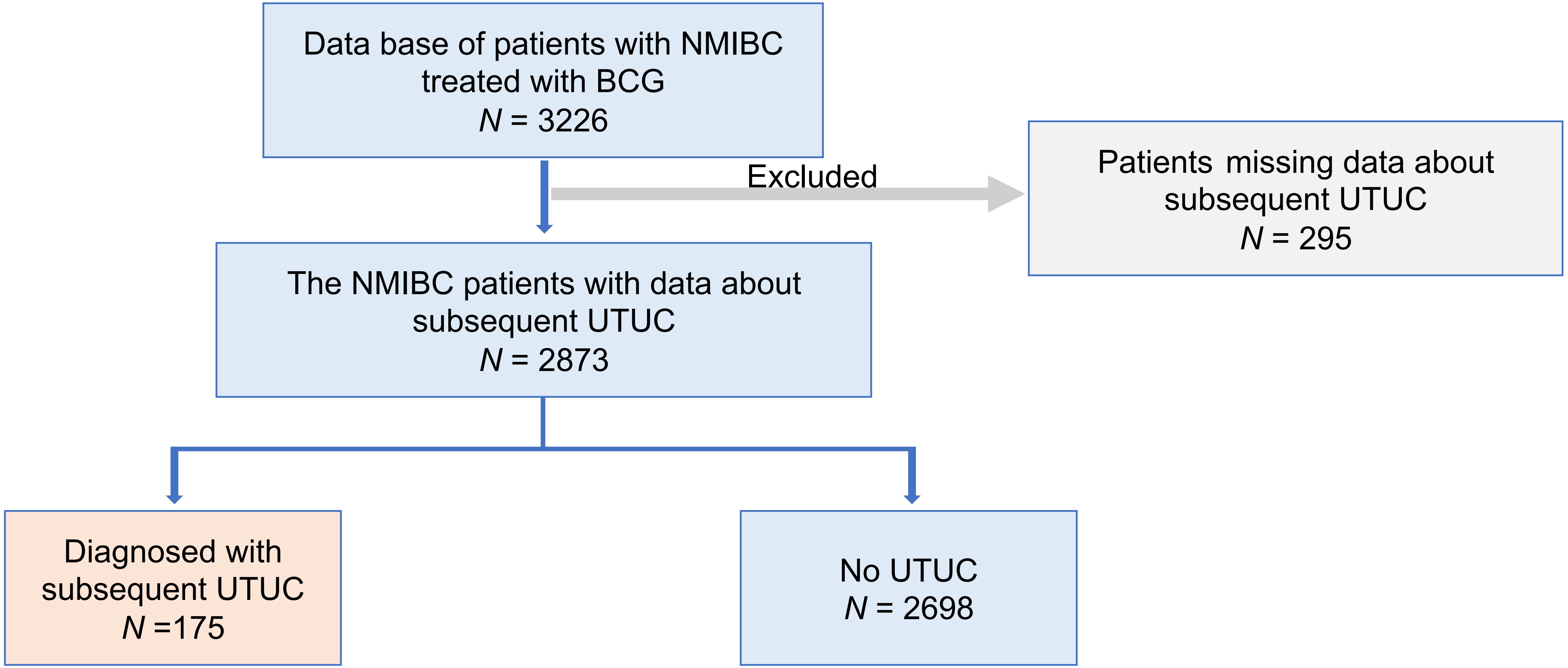

NMIBC, non-muscle invasive bladder cancer ; BCG, bacillus Calmette-Guerin ; UTUC, upper urinary tract carcinoma
